# Cyclic Soft Groups and Their Applications on Groups

**DOI:** 10.1155/2014/437324

**Published:** 2014-07-15

**Authors:** Hacı Aktaş, Şerif Özlü

**Affiliations:** ^1^Department of Mathematics, Faculty of Science, Erciyes University, 38039 Kayseri, Turkey; ^2^Kilis 7 Aralık University, 79000 Kilis, Turkey

## Abstract

In crisp environment the notions of order of group and cyclic group are well known due to many applications. In this paper, we introduce order of the soft groups, power of the soft sets, power of the soft groups, and cyclic soft group on a group. We also investigate the relationship between cyclic soft groups and classical groups.

## 1. Introduction

Most of our real life problems in economics, engineering, environment, social science, and medical science involve imprecise data that contain uncertainties. To solve these kinds of problems, it is quite difficult to successfully use classical methods. However, there are some well-known theories (probability, fuzzy sets [1], vague sets [2], rough sets [3], etc.) which can be considered as mathematical tools for dealing with uncertainties. All these theories have their inherent difficulties pointed out by Molodtsov [4].

The theory of soft sets, proposed by Moldtsov [4], is an extension of set theory for the study of intelligent systems characterized by insufficient and incomplete information. His pioneer paper has undergone tremendous growth and applications in the last few years. Maji et al. [5] give an application of soft set theory in a decision making problem by using the rough sets and they conducted a theoretical study on soft sets in a detailed way [6]. Chen et al. [7] proposed a reasonable definition of parameterizations reduction of soft sets and compared them with the concept of attributes reduction in rough set theory.

The algebraic structures of set theories which deal with uncertainties have been studied by some authors. Rosenfeld [8] proposed fuzzy groups to establish results for the algebraic structures of fuzzy sets. Rough groups are defined by Biswas and Nanda [9], and some others (i.e., Bonikowaski [10] and Iwinski [11]) studied algebraic properties of rough sets. Fuzzification of algebraic structures was studied by many authors [8, 12, 13].

Many papers on soft algebras have been published since Aktaş and Ça$\overset{˘}{\text{g}}$man [14] introduced the notion of a soft group in 2007. Recently, Jun et al. [15] studied soft ideals and idealistic soft BCK/BCI-algebras. Acar et al. [16] introduced initial concepts of soft rings. Aygünoğlu and Aygün [17] introduced the concept of fuzzy soft group and, in the meantime, they studied its properties and structural characteristics. Atagün and Sezgin [18] introduced and studied the concepts of soft subrings, soft ideal of a ring, and soft subfields of a field.

Our interest, in this paper, is to define order of the soft group and cyclic soft group by using definition of the soft group that was defined in [14]. We then find out the relationships between cyclic soft groups and classical groups. Finally, we conclude the study with suggestions for future work.

## 2. Preliminaries

The following definitions and preliminaries are required in the sequel of our work and they are presented in brief.

Throughout this work, *U* is an initial universe set, *E* is a set of parameters, *P*(*U*) is the power set of *U*, *A* ⊂ *E*, and *G* denotes a group with identity *e*.

**Definition 1 (see [4]).**

A pair (*F*, *A*) is called a soft set over *U*, where *F* is a mapping given by

(1) $$\begin{array}{c} F:A⟶P\left(U\right). \end{array}$$

In other words, a soft set over *U* is a parameterized family of subsets of the universe *U*.

**Definition 2 (see [6]).**

For two soft sets (*F*, *A*) and (*G*, *B*) over *U*, (*F*, *A*) is called a soft subset of (*G*, *B*), if

1. *A* ⊂ *B* and
2. ∀*ɛ* ∈ *A*; *F*(*ɛ*) and *G*(*ɛ*) are identical approximations.

It is denoted by $\left(F,A)\tilde{\subset }(G,B\right)$.

(*F*, *A*) is called a soft superset of (*G*, *B*) if (*G*, *B*) is a soft subset of (*F*, *A*). It is denoted by $\left(F,A)\tilde{⊃}(G,B\right)$.

**Definition 3 (see [6]).**

If (*F*, *A*) and (*G*, *B*) are two soft sets, then (*F*, *A*) AND (*G*, *B*) is denoted (*F*, *A*)∧(*G*, *B*). (*F*, *A*)∧(*G*, *B*) is defined as (*H*, *A* × *B*), where *H*(*α*, *β*) = *F*(*α*)∩*G*(*β*), for all (*α*, *β*) ∈ *A* × *B*.

**Definition 4 (see [6]).**

If (*F*, *A*) and (*G*, *B*) are two soft sets, then (*F*, *A*) OR (*G*, *B*), denoted by (*F*, *A*)∨(*G*, *B*), is defined by (*F*, *A*)∨(*G*, *B*) = (*H*, *A* × *B*), where *H*(*α*, *β*) = *F*(*α*) ∪ *G*(*β*), ∀(*α*, *β*) ∈ *A* × *B*.

**Definition 5 (see [6]).**

Union of two soft sets of (*F*, *A*) and (*G*, *B*) over *U* is the soft set (*H*, *C*), where *C* = *A* ∪ *B* and ∀*e* ∈ *C*,

(2) $$\begin{array}{c} H\left(e\right)=\{\begin{array}{cc} F\left(e\right), & \text{if}\;e\in A-B,\\ G\left(e\right), & \text{if}\;e\in B-A,\\ F\left(e\right)\cup G\left(e\right), & \text{if}\;e\in A\cap B. \end{array} \end{array}$$

It is denoted by $\left(F,A)\tilde{\cup }(G,B)=(H,C\right)$.

**Definition 6 (see [14]).**

Let (*F*, *A*) be a soft set over *G*. Then (*F*, *A*) is said to be a soft group over *G* if and only if *F*(*x*) is subgroup of *G* for all *x* ∈ *A*.

**Definition 7 (see [14]).**

One considers the following.

1. (*F*, *A*) is said to be an identity soft group over *G* if *F*(*x*) = {*e*} for all *x* ∈ *A*, where *e* is the identity element of *G*.
2. (*F*, *A*) is said to be an absolute soft group over *G* if *F*(*x*) = *G* for all *x* ∈ *A*.

**Definition 8 (see [14]).**

Let (*F*, *A*) and (*H*, *K*) be two soft groups over *G*. Then (*H*, *K*) is a soft subgroup of (*F*, *A*), written as $\left(H,K)\tilde{<}(F,A\right)$, if

1. *K* ⊂ *A*,
2. *H*(*x*) is a subgroup of *F*(*x*) for all *x* ∈ *K*.

**Definition 9 (see [14]).**

Let (*F*, *A*) and (*H*, *B*) be two soft groups over *G* and *K*, respectively, and let *f* : *G* → *K* and *g* : *A* → *B* be two functions. Then one says (*f*, *g*) is a soft homomorphism and (*F*, *A*) is soft homomorphic to (*H*, *B*), denoted by (*F*, *A*)~(*H*, *B*), if the following conditions are satisfied:

1. *f* is a homomorphism from *G* onto *K*,
2. *g* is a mapping from *A* onto *B*,
3. *f*(*F*(*x*)) = *H*(*g*(*x*)) for all *x* ∈ *A*.

In this definition, if *f* is an isomorphism from *G* to *K* and *g* is a one-to-one mapping from *A* onto *B*, then we say that (*f*, *g*) is a soft isomorphism and (*F*, *A*) is soft isomorph to (*H*, *B*) which is denoted by (*F*, *A*)≃(*H*, *B*). The image of soft group (*F*, *A*) under soft homomorphism (*f*, *g*) will be denoted by (*f*(*F*), *g*(*A*)).

**Definition 10 (see [14]).**

Let (*F*, *A*) and (*H*, *B*) be two soft groups over *G* and *K*, respectively. The product of soft groups (*F*, *A*) and (*H*, *B*) is defined as (*F*, *A*)×(*H*, *B*) = (*U*, *A* × *B*), where *U*(*x*, *y*) = *F*(*x*) × *H*(*y*), for all (*x*, *y*) ∈ *A* × *B*.

## 3. The Order of Soft Groups

Since the elements of a cyclic group are the powers of the element, properties of cyclic groups are closely related to the properties of the powers of an element. In this paper, we define order of the soft group by using definition of the soft group that was defined in [14]. We then investigate their properties.

**Definition 11 .**

A pair (*F*, *A*) is called surjective soft set over *U*, where *F* is a surjective mapping given by *F* : *A* → *P*(*U*).

Throughout this study, *G* denotes a group and the soft set (*F*, *A*) will be a surjective soft set. The element *F*(*a*) is used instead of the element (*a*, *F*(*a*)) of (*F*, *A*).

**Definition 12 .**

Let (*F*, *A*) be a soft set over *G* and *F*(*x*)∈(*F*, *A*) for *x* ∈ *A*. Then *F*(*x*)^*n*^ = {*a* ^*n*^ : *a* ∈ *F*(*x*), *n* ∈ *Z*} is called *n*-power of *F*(*x*).

**Example 13 .**

Let (*F*, *A*) be a soft set over *S*_3_, where *A* = *S*_3_, and let

(3) (F,A)={F(e)={e},F(12)={e,(12)}, F(13)={e,(13)},F(23)={e,(23)}, F(123)={e,(123),(132)}}

be a soft set over group *S*_3_. And the third power of *F*(123) is *F*(123)^3^ = {*e* ^3^, (123)^3^, (132)^3^} = {*e*, *e*, *e*} = {*e*}.

Of course, when the group is additive, the *n*th power of *F*(*x*) will be written by *nF*(*x*) = {*na* : *a* ∈ *F*(*x*), *n* ∈ *Z*}.

**Theorem 14 .**

Let (*F*, *A*) be a soft set over *G* and *F*(*x*), *F*(*y*)∈(*F*, *A*) for *x*, *y* ∈ *A*. Then, for all *n* ∈ *Z*,

1. (*F*(*x*)∩*F*(*y*))^*n*^⊆*F*(*x*)^*n*^∩*F*(*y*)^*n*^, for all *n* ∈ *Z*,
2. (*F*(*x*) ∪ *F*(*y*))^*n*^ = *F*(*x*)^*n*^ ∪ *F*(*y*)^*n*^, for all *n* ∈ *Z*,
3. (*F*(*x*) × *F*(*y*))^*n*^ = *F*(*x*)^*n*^ × *F*(*y*)^*n*^, for all *n* ∈ *Z*.

**Proof**

Let *a* ^*n*^ ∈ (*F*(*x*)∩*F*(*y*))^*n*^, for *n* ∈ *Z*. From Definition 12 *a* ∈ *F*(*x*)∩*F*(*y*) and *a* ^*n*^ ∈ *F*(*x*)^*n*^ and *a* ^*n*^ ∈ *F*(*y*)^*n*^. This means that *a* ^*n*^ ∈ *F*(*x*)^*n*^∩*F*(*y*)^*n*^. This completes the proof.  Theorem 14(2) and Theorem 14(3) can be proved similarly by using Definition 12.

In general, the opposite of Theorem 14(1) is not true. We illustrate an example of this situation.

**Example 15 .**

Let *A* = {0,1} and let *F* : *A* → *P*(*Z*) be a function such that *F*(0) = {2*k* : *k* ∈ *Z*} and *F*(1) = {2*k* + 1 : *k* ∈ *Z*}. The intersection is *F*(0)∩*F*(1) = *∅*. So (*F*(0)∩*F*(1))^2^ = *∅*. On the other hand *F*(0)^2^∩*F*(1)^2^ ≠ *∅*. Consequently (*F*(*x*)∩*F*(*y*))^*n*^ ≠ *F*(*x*)^*n*^∩*F*(*y*)^*n*^.

**Definition 16 .**

Let (*F*, *A*) be a soft set over *G* and *F*(*x*)∈(*F*, *A*). If there is a positive integer *n* such that *F*(*x*)^*n*^ = {*e*}, then the least such positive integer *n* is called the order of *F*(*x*). If no such *n* exists, then *F*(*x*) has infinite order. The order of *F*(*x*) is denoted by |*F*(*x*)|.

If (*F*, *A*) is a soft group over *G*, then the order of *F*(*x*)∈(*F*, *A*) coincides with the order of *F*(*x*), which is subgroup of *G*. Of course if there is any element *x* in *A* such that *F*(*x*) = {*e*}, then the order of *F*(*x*) is 1.

**Example 17 .**

In Example 13 the order of element *F*(123) is 3.

Let (*F*, *N*) be a soft group over group of integer numbers *Z*, where *F* is a mapping from natural numbers *N* to *P*(*Z*) such that *F*(*n*) = *nZ* for all *n* ∈ *N*. There is no any positive integer *m* such that *F*(*n*)^*m*^ = {0}, so *F*(*n*) has infinite order for all *n* ∈ *N* − {0}.

**Theorem 18 .**

Let *G* be finite group and (*F*, *A*) a soft group over *G*. Then, the orders of elements of (*F*, *A*) are finite.

**Proof**

It is straightforward.

**Theorem 19 .**

Let (*F*, *A*) be a soft set over finite group *G* and *F*(*x*)∈(*F*, *A*) for *x* ∈ *A*. Then, the order of *F*(*x*) is the least common multiple (LCM) of order of elements of *F*(*x*).

**Proof**

Let *n* be the order of *F*(*x*). Then *F*(*x*)^*n*^ = {*e*}. This means that *a* ^*n*^ = *e* for all *a* ∈ *F*(*x*). We know from classical group theory that |*a*|∣*n*, namely, *a*, divides *n* for all *a* ∈ *F*(*x*). Thus, *n* is common multiple of elements of *F*(*x*). Let *m* be another common multiple of elements of *F*(*x*). Then, by reason of *a* ^*m*^ = *e* for all *a* ∈ *F*(*x*), *F*(*x*)^*m*^ = {*e*}. However, since *n* is the least number that satisfies the condition *F*(*x*)^*n*^ = {*e*}, hence *n*∣*m*. This completes the proof.

**Theorem 20 .**

Let *G* be finite group, (*F*, *A*) a soft group over *G*, and *F*(*x*) and *F*(*y*) the elements of (*F*, *A*). Then, for all *x*, *y* ∈ *A*, one has the following:

1. |*F*(*x*)∩*F*(*y*)| ≤ *GCD*(|*F*(*x*)|, |*F*(*y*)|) for *x*, *y* ∈ *A*,
2. |*F*(*x*) ∪ *F*(*y*)| = *LCM*(|*F*(*x*)|, |*F*(*y*)|) for *x*, *y* ∈ *A*,
3. |*F*(*x*) × *F*(*y*)| = |*F*(*x*)||*F*(*y*)| for *x*, *y* ∈ *A*.

**Proof**

We consider the following.

1. *F*(*x*)∩*F*(*y*) is subgroup of *F*(*x*) and *F*(*y*), so |*F*(*x*)∩*F*(*y*)|∣|*F*(*x*)| and |*F*(*x*)∩*F*(*y*)|∣|*F*(*y*)|. It follows |*F*(*x*)∩*F*(*y*)| ≤ *GCD*(|*F*(*x*)|, |*F*(*y*)|).
2. Let |*F*(*x*) ∪ *F*(*y*)| = *k*, |*F*(*x*)| = *m*, and |*F*(*y*)| = *n*. From Theorem 14(*F*(*x*) ∪ *F*(*y*))^*k*^ = *F*(*x*)^*k*^ ∪ *F*(*y*)^*k*^ = {*e*}. This follows *n*∣*k* and *m*∣*k*. Thus *k* is a common multiple of *m* and *n*. Let *t* be another common multiple of *m* and *n*. Consider (*F*(*x*)∩*F*(*y*))^*t*^ = *F*(*x*)^*t*^ ∪ *F*(*y*)^*t*^ = {*e*} ∪ {*e*} = {*e*}. Since *k* is the least positive integer that satisfied the condition (*F*(*x*) ∪ *F*(*y*))^*k*^ = {*e*}, *k* divides *t*. Hence *k* is LCM order of *F*(*x*) and *F*(*y*). This completes the proof.
3. Since *F*(*x*) and *F*(*y*) are subgroups of *G*, it is seen easily.

**Definition 21 .**

Let *G* be group and (*F*, *A*) soft set over *G*. The set

(4) $$\begin{array}{c} {\left(F,A\right)}^{n}=\left\{F{\left(x\right)}^{n}:x\in A,n\in Z\right\} \end{array}$$

is called *n*th power of soft set (*F*, *A*).

**Example 22 .**

Let (*F*, *A*) be a soft set over *S*_3_ defined in Example 13. Then, the second power of (*F*, *A*) is that (*F*, *A*)^2^ = {*F*(*e*)^2^ = *F*(*e*), and *F*(12)^2^ = *F*(*e*),  *F*(13)^2^ = *F*(*e*),  *F*(23)^2^ = *F*(*e*),  *F*(123)^2^ = *F*(132)}.

**Theorem 23 .**

Let (*F*, *A*) and (*E*, *B*) be two soft sets over *G*. Then,

1. ((*F*, *A*)∨(*E*, *B*))^*n*^ = (*F*, *A*)^*n*^∨(*E*, *B*)^*n*^,
2. if *A*⊆*B* and, for all *a* ∈ *A*, *F*(*a*) and *E*(*a*) are identical approximations, then ((*F*, *A*)∧(*E*, *B*))^*n*^⊆(*F*, *A*)^*n*^∧(*E*, *B*)^*n*^.

**Proof**

We consider the following.

(1) Suppose that (*F*, *A*)∨(*E*, *B*) = (*H*, *A* × *B*) and (*F*, *A*)^*n*^∨(*E*, *B*)^*n*^ = (*T*, *A* × *B*). We can write ((*F*, *A*)∨(*E*, *B*))^*n*^ = (*H*, *A* × *B*)^*n*^. Using Definition 21 and Theorem 14 we have
  (5) (H,A×B)n={H(a,b)n:(a,b)∈A×B}={(F(a)∪E(b))n:(a,b)∈A×B}={F(a)n∪E(b)n:(a,b)∈A×B}={T(a,b):(a,b)∈A×B}=(F,A)n∨(E,B)n.
(2) Suppose that (*F*, *A*)∧(*E*, *B*) = (*H*, *A* × *B*) and (*F*, *A*)^*n*^∧(*E*, *B*)^*n*^ = (*T*, *A* × *B*). Using the same arguments in (1), we have
  (6) (H,A×B)n={H(a,b)n:(a,b)∈A×B}={(F(a)∩E(b))n:(a,b)∈A×B}⊆{F(a)n∩E(b)n:(a,b)∈A×B}={T(a,b):(a,b)∈A×B}=(F,A)n∧(E,B)n.

If (*F*, *A*) and (*E*, *B*) are both soft groups, then, for all *a*, *b* ∈ *A*,  *F*(*a*) and *F*(*b*) are all subgroups of *G*, and so *F*(*a*) and *E*(*b*) contain the identity element *e* of *G*. Thus, the set *F*(*a*)∩*E*(*b*) contains at least *e*; hence (*F*(*a*)∩*E*(*b*))^*n*^ ≠ *∅*. It means that if we take in Theorem 23(2) (*F*, *A*) and (*E*, *B*) as soft groups, not soft sets, then the extra condition can be added in Theorem 23(2).

In classical groups, the order of a group is defined as the number of elements it contains. But in soft groups, it differs from classical groups.

**Definition 24 .**

Let (*F*, *A*) be soft group over *G*. If *G* is a finite group, then the least common multiple of orders of elements of (*F*, *A*) is called order of (*F*, *A*). If *G* is an infinite group, then the order of (*F*, *A*) is defined to be the number of elements of (*F*, *A*) and the order of soft group (*F*, *A*) is denoted by |(*F*, *A*)|.

Of course, if (*F*, *A*) is surjective, then the number of elements of (*F*, *A*) is the number of elements in *A*.

**Example 25 .**

In Example 13 the order of (*F*, *A*) is 6 and in Example 17 if we chose *N* = *N*_4_ = {0,1, 2,3, 4} and *F*(*n*) = *nZ*, for *n* ∈ *N*_4_, then the order of soft group (*F*, *N*_4_) is 5.

We give the following results similar to Lagrange Theorem in group theory.

**Theorem 26 .**

Let (*F*, *A*) be a soft group over a finite group *G* and *F*(*x*)∈(*F*, *A*). Then one has the following.

1. The order of *F*(*x*) divides the order of (*F*, *A*). In particular, *F*(*x*)^|(*F*,*A*)|^ = {*e*}.
2. The order of (*F*, *A*) divides the order of *G*.

When *G* is a finite group, then the order (*F*, *A*) is a finite and the orders of elements of (*F*, *A*) divide the order of (*F*, *A*). However when *G* is an infinite group, then the order of (*F*, *A*) can be finite or infinite. Hence, Theorem 26 is not true for infinite groups.

**Theorem 27 .**

Let *G* be a finite group and let (*F*, *A*) and (*E*, *B*) be two soft groups over *G*. Then |(*F*, *A*)∧(*E*, *B*)| ≤ |(*F*, *A*)| and |(*F*, *A*)∧(*E*, *B*)| ≤ |(*E*, *B*)|.

**Proof**

Suppose that (*F*, *A*)∧(*E*, *B*) = (*H*, *C*), where *C* = *A* × *B*. Using fundamental theorems and definitions in group theory and Definitions 24 and 3, we have

(7) |(F,A)∧(E,B)|=LCM(H(ai,bi)),for(ai,bi)∈A×B=LCM(F(ai)∩E(bi))≤LCM(F(ai))=|(F,A)|.

The other inequality is shown similarly.

## 4. Cyclic Soft Groups

The class of cyclic groups is an important class in group theory. In this section, we study soft groups which are generated by one element of *P*(*G*). We define cyclic soft group and prove some of their properties which are analogous to the crisp case.

**Definition 28 .**

Let (*F*, *A*) be a soft group over *G* and *X* an element of *P*(*G*). The set {(*a*, 〈*x*〉) : *F*(*a*) = 〈*x*〉, *x* ∈ *X*} is called a soft subset of (*F*, *A*) generated by the set *X* and denoted by 〈*X*〉. If (*F*, *A*) = 〈*X*〉, then the soft group (*F*, *A*) is called the cyclic soft group generated by *X*.

If (*F*, *A*) is a cyclic soft group over *G*, then we can write it in this form (*F*, *A*) = {*F*(*a*) = 〈*x*〉 : *a* ∈ *A*, *x* ∈ *G*}, where {*x* ∈ *G*} is element of *P*(*G*). That is to say, if all the elements of (*F*, *A*) are generated by any elements of *X* of *P*(*G*), then (*F*, *A*) is a cyclic soft group over *G*.

If *G* is a cyclic group, then (*F*, *A*) is a soft cyclic group over *G* since all subgroups of cyclic group are cyclic but the reverse is not always true.

As an illustration, let us consider the following example.

**Example 29 .**

Let *G* = *S*_3_ be the symmetric group and *A* = {*e*, (12), (13), (23), (123)} the set of parameters. If we construct a soft set (*F*, *A*) over *G* such that *F*(*x*) = {*y* ∈ *G* : *y* = *x* ^*n*^, *n* ∈ *Z*} for all *x* ∈ *A*, then one can easily show that (*F*, *A*) is a soft cyclic group over *G*; however *G* is not a cyclic group.

In the following theorem, we can give some properties of cyclic soft groups that has similar features of the classical cyclic groups.

**Theorem 30 .**

One considers the following.

1. If (*F*, *A*) is a finite cyclic soft group generated by *X*, then |(*F*, *A*)| = *LCM*(|*x*_*i*_|), where *x*_*i*_ ∈ *X*.
2. If (*F*, *A*) is an infinite cyclic soft group generated by *X*, then |(*F*, *A*)| = |*X*|.
3. If (*F*, *A*) is an identity soft group, then it is a cyclic soft group generated by {*e*}.
4. Let (*F*, *A*) be an absolute soft group defined on *G*. Then, (*F*, *A*) is a cyclic soft group if and only if *G* is a cyclic group.
5. Let (*F*, *A*) be a soft group on *G*. If the order of *G* is prime, then (*F*, *A*) is a cyclic soft group.
6. A soft subgroup of a cyclic soft group is cyclic soft group.

**Proof**

It is easily seen from Definitions 24, 7, and 8.

**Theorem 31 .**

Let (*f*, *g*) be a soft homomorphism of the soft group (*F*, *A*) over *G* into the soft group (*H*, *B*) over *K*. If (*F*, *A*) is a cyclic soft group over *G*, then (*f*(*F*), *g*(*A*)) is a cyclic soft group over *K*.

**Proof**

First, we show that (*f*(*F*), *g*(*A*)) is a soft group over *K*. Since *f* is a homomorphism from *G* to *K*, *f*(*F*(*x*)) = *H*(*g*(*x*)) is a subgroup of *K* for all *g*(*x*) ∈ *g*(*A*). Thus (*f*(*F*), *g*(*A*)) is a soft group over *K*. Since *F*(*x*) is cyclic subgroup for all *x* in *A*, image of *F*(*x*) under *f* is cyclic; that is, *f*(*F*(*x*)) = *H*(*g*(*x*)) is cyclic subgroup of *K* for all *g*(*x*) ∈ *g*(*A*). Consequently, (*f*(*F*), *g*(*A*)) is a cyclic soft group over *K*.

**Theorem 32 .**

Let (*F*, *A*) and (*H*, *B*) be two soft isomorphic soft groups over *G* and *K*, respectively. If (*F*, *A*) is a cyclic soft group, then so is (*H*, *B*).

**Proof**

First of all note that since (*F*, *A*) is a soft isomorphic to (*H*, *B*), there is an *f* homomorphism from *G* to *K* such that *f*(*F*(*x*)) = *H*(*g*(*x*)) for all *x* ∈ *A*, where *g* is a one-to-one mapping from *A* onto *B*. Since *F*(*x*) is a cyclic subgroup for all *x* in *A* and *f* is a homomorphism, then *H*(*g*(*x*)) is a cyclic subgroup of *K*. Thus, all elements of (*H*, *B*) are cyclic. This result completes the proof.

**Theorem 33 .**

If (*F*, *A*) and (*H*, *B*) are two cyclic soft groups over *G*, then (*F*, *A*)∧(*H*, *B*) is a cyclic soft group over *G*.

**Proof**

Let (*F*, *A*)∧(*H*, *B*) = (*E*, *A* × *B*), where *E*(*α*, *β*) = *F*(*α*)∩*H*(*β*), ∀(*α*, *β*) ∈ *A* × *B*. Since *F*(*α*) and *H*(*β*) are cyclic subgroups of *G* for all *α* ∈ *A* and *β* ∈ *B* and *F*(*α*)∩*H*(*β*) is a subgroup of both *F*(*α*) and *H*(*β*), *F*(*α*)∩*H*(*β*) is cyclic subgroup of *G* for all (*α*, *β*) ∈ *A* × *B*. Hence, (*H*, *A* × *B*) is cyclic soft group over *G*.

**Theorem 34 .**

Let (*F*, *A*) and (*H*, *B*) be two cyclic soft groups over *G* and *A*∩*B* = *∅*. Then, $\left(F,A)\tilde{\cup }(H,B\right)$ is a cyclic soft group over *G*.

**Proof**

It is trivial.

**Theorem 35 .**

Let (*F*, *A*) and (*H*, *B*) be two cyclic soft groups of finite orders *m* and *n* over *G* and *K*, respectively. If *m* and *n* are relatively prime, then the product (*F*, *A*)×(*H*, *B*) is a cyclic soft group.

**Proof**

Let (*m*, *n*) = 1. According to Lagrange Theorem, |*F*(*x*)| divides *m* and |*H*(*y*)| divides *n* for all *x* ∈ *A* and for all *y* ∈ *B*. Since (*m*, *n*) = 1, |*F*(*x*)| and |*H*(*y*)| are relatively prime. So *F*(*x*) × *H*(*y*) is cyclic group for all (*x*, *y*) ∈ *A* × *B*. This completes the proof.

## 5. Conclusion

In this paper, we have expanded the soft set theory. We have focused on order of the soft groups and investigated relationship between the order of soft groups and the order of classical groups. Additionally, we have studied the algebraic properties of cyclic soft groups with respect to a group structure. Our future work will focus on the relationships between cyclic soft groups and other algebraic structures such as rings and fields.
